# Clinical Characteristics of SARS-CoV-2 Acute Pulmonary Embolism and Adjusted D-dimer for Emergency Department Patients

**DOI:** 10.5811/westjem.58619

**Published:** 2023-10-25

**Authors:** Iltifat Husain, James C. O’Neill, Jacob H. Schoeneck, K. Alexander Soltany, Hollins Clark, Erika Weidman Rice, Alex Gross, Jonathan Redding, David M. Cline

**Affiliations:** * Wake Forest School of Medicine, Department of Emergency Medicine, Winston-Salem, North Carolina; † University of North Carolina Chapel Hill, Department of Radiology, Chapel Hill, North Carolina

## Abstract

**Introduction:**

Severe acute respiratory syndrome coronavirus 2 (SARS-CoV-2) and acute pulmonary embolism (APE) present a diagnostic challenge in the emergency department (ED) setting. We aimed to identify key clinical characteristics and D-dimer thresholds associated with APE in SARS-CoV-2 positive ED patients.

**Methods:**

We performed a multicenter, retrospective cohort study for adult patients who were diagnosed with coronavirus 2019 (COVID-19) and had computed tomography pulmonary angiogram (CTPA) performed between March 17, 2020–January 31, 2021. We performed univariate analysis to determine numeric medians, chi-square values for association between clinical characteristic and positive CTPA. Logistic regression was used to determine the odds of a clinical characteristic being associated with a diagnosis of APE.

**Results:**

Of 408 patients who underwent CTPA, 29 (7.1%) were ultimately found to have APE. In multivariable analysis, patients with a body mass index greater than 32 (odds ratio [OR] 4.4, 95% confidence interval [CI] 1.0 -19.3), a heart rate greater than 90 beats per minute (bpm) (OR 5.0, 95% CI 1.0-24.9), and a D-dimer greater than 1,500 micrograms per liter (μg/L) (OR 5.6, 95% CI 1.6-20.2) were significantly associated with pulmonary embolism. In our population that received a D-dimer and was SARS-CoV-2 positive, limiting CTPA to patients with a heart rate over 90 or a D-dimer value over 1500 μg/L would reduce testing 27.2% and not miss APE.

**Conclusion:**

In patients with acute COVID-19 infections, D-dimer at standard cutoffs was not usable. Limiting CTPA using a combination of heart rate greater than 90 bpm or D-dimer greater than 1,500 μg/L would significantly decrease imaging in this population.

Population Health Research Capsule What do we already know about this issue?

*There is an increased incidence of acute pulmonary embolism (APE) in patients hospitalized with COVID-19.*

What was the research question?

*Can D-dimer thresholds and clinical characteristics of ED patients be used to determine whether computed tomography pulmonary angiography (CTPA) is indicated to rule out APE?*

What was the major finding of the study?

*We found that performing CTPA on patients with a heart rate >90 and a D-dimer value over 1,500 μg/L had a sensitivity of 100% (95% CI 80–100%) and would reduce testing 27.2% while being unlikely to miss APE.*

How does this improve population health?

*In treating COVID-19 patients with suspected APE, emergency physicians should use different D-dimer thresholds in conjunction with patients’ heart rates.*

## INTRODUCTION

Since the emergence of severe acute respiratory syndrome coronavirus 2 (SARS-CoV-2) in Wuhan, China, there have been over one million deaths and over 89 million cases related to coronavirus disease 2019 (COVID-19) in the United States.^1^ Although COVID-19 was initially characterized as a respiratory illness, critically ill patients have proven to have an associated hypercoagulable state.^2^ The hypercoagulable state appears to originate in the pulmonary vasculature and evolves into a generalized hypercoagulable state resulting in macro- and microvascular thrombosis such as acute pulmonary embolism (APE).^3^ ^,^ ^4^

While growing research has documented the incidence of APE in hospitalized patients with COVID-19, few published studies have evaluated patients with SARS-CoV-2 infection and associated diagnosis of APE upon initial presentation to the emergency department (ED). Previous studies of APE risk in ED COVID-19 patients have been either inconclusive and even contradictory.^5^ ^–^ ^8^ The limited information suggests that rates of APE in non-hospitalized COVID-19 patients may be as high as 18%, more than seven-fold higher than in the non-COVID-19 ED population.^9^ ^,^ ^10^ With conflicting evidence on the incidence of APE in the ED setting, there remains a paucity of literature discussing diagnostic algorithms and computed tomography pulmonary angiogram (CTPA) diagnostic yield (the percentage of positive scans) for APE in ED COVID-19 patients. Proposed algorithms using D-dimer levels vary greatly and are not ED-specific.^8^

The diagnosis of APE in COVID-19 patients presents a diagnostic dilemma in the ED. The post-acute SARS-CoV-2 symptoms of dyspnea, chest pain, and tachycardia are all associated with clinical characteristics for APE.^8^ Further, traditional methods of ruling out APE, such as using D-dimer in low-risk patients, are not feasible because D-dimer levels are commonly elevated in COVID-19 patients.^9^ In particular, a known relationship exists between the level of D-dimer elevation and COVID-19 severity.^10^

In this derivation study, our primary objective was to identify which of the commonly known risk factors for APE were associated with APE in a COVID-19 patient population in the ED. Our secondary objective was to identify D-dimer values associated with APE in the ED setting.

## METHODS

This retrospective review was approved by our institutional review board. We performed a multicenter, retrospective cohort analysis for adult patients who arrived to any of the five EDs within the Atrium Health Wake Forest Baptist system between March 17, 2020–January 31, 2021. The EDs included one academic medical center and four regional community hospitals.

The inclusion criteria for the study were patients >16 years of age who tested positive for SARS-CoV-2 or had a COVID-19-related diagnosis and had a CTPA study ordered. A COVID-19-related diagnosis was based on International Classification of Diseases, 10^th^ Rev, (ICD-10) codes. (The list of ICD-10 codes used is included in Appendix 1.) Using these criteria, we extracted a patient list from our electronic health record (EHR) via a health analytics software and services company (Roundtable Analytics, Research Triangle Park, NC). Final inclusion was based on confirmation of SARS-CoV-2 based on reverse transcription polymerase chain reaction (RT-PCR) or rapid antigen testing. This article follows the Strengthening and Reporting of Observational Studies in Epidemiology (STROBE) guidelines.^11^

The clinical characteristics we focused on were based on commonly used, ED-specific APE decision rules: pulmonary embolism rule-out criteria, Well’s criteria for APE, and the Geneva Score for APE.^12^ ^–^ ^14^ D-dimer values consisted of both fibrinogen equivalent unit (FEU) and D-dimer unit (DDU). To bring parity to the different assays, DDU results were doubled. This was performed in a manner that has been described in prior COVID-19 D-dimer studies.^15^ The cut-off for one hospital’s FEU assay was 399 micrograms per liter (μg/L), while the other FEU assay cut-offs were 500 μg/L. The cut-off for the DDU assay was 230 μg/L. We calculated chi-square values, odds ratios (OR) with 95% confidence intervals (CI), and Kruskal-Wallis testing of numeric medians, and we used logistic regression to compare characteristics of patients who had APE to those who did not, using *P* < 0.05 as significant.

## RESULTS

We identified 425 patients who underwent evaluation for pulmonary embolism with CTPA in the setting of a suspected COVID-19 infection during the study period. Of this cohort, 408 patients (96%) tested positive for SARS-CoV-2 infection by RT-PCR analysis or rapid antigen testing and were included in our study. Of the CTPAS performed, 72% were done at the four community hospitals and 28% at the academic medical center hospital. Patient demographic and clinical characteristics are summarized in Table 1. Twenty-nine patients (7.1%) were ultimately found to have an APE on CTPA. The diagnostic yield of APE on CTPA varied from a high of 9.9% at one of the medium-sized community hospitals to a low of 2.3% at the smallest community hospital in our system.

**Table 1.** Characteristics of patient population.

Clinical characteristics	Total N = 408 (%)
Female	224 (54.9)
Male	184 (45.1)
Age	
18–49	126 (30.9)
50–69	169 (41.4)
70+	113 (27.7)
Race/Ethnicity	
White, non-Hispanic	262 (64.2)
Non-White, non-Hispanic	116 (28.4)
Hispanic	30 (7.4)
Clinical features	
Hemoptysis	13 (3.51)
Leg swelling	2 (0.50)
Past history of DVT	24 (6.50)
History of malignancy	11 (2.7)
Estrogens	16 (3.9)
Recent surgery	8 (1.9)
No anticoagulation	273 (66.9)
Aspirin	91 (22.3)
DOAC	15 (3.7)
Warfarin	1 (0.2)
Clopidogrel	4 (1.0)
Two or more anticoagulants	24 (5.9)
Symptom severity	
Asymptomatic	2 (0.5)
Mild	58 (14.2)
Moderate	203 (49.8)
Severe	145 (35.5)
APE on CTPA study	
Positive	29 (7.1)
Negative	379 (92.9)
Heart rate (HR) bpm	
HR ≥90	251 (63.1)
HR<90	147 (36.9)
HR ≥100	223 (56.0)
HR <100	175 (44.0)
Oxygen saturation	
<90	66 (16.1)
<95	156 (38.2)
Supplemental oxygen requirement	170 (41.6)
No supplemental oxygen	238 (58.4)
Date of illness	
0–4	132 (33.7)
5–10	115 (29.3)
>10	145 (37.0)
Excluded no data	5
BMI	
<25	65 (16.4%)
Overweight (BMI 25–29.9)	99 (24.8%)
Obesity (BMI ≥30)	234 (58.8%)

*DVT*, deep vein thrombosis; *DOAC*, direct oral anticoagulants; *APE*, acute pulmonary embolism; *CTPA*, computed tomography pulmonary angiogram; *bpm*, beats per minute; *BMI*, body mass index.

The heart rate was significantly higher in patients who were found to have APE (median 102 beats per minute [bpm], interquartile range [IQR] 23 compared to 95 bpm (IQR 28), *P* = 0.0133]. Patients with APE were significantly more likely to present with hypoxia or a supplemental oxygen requirement (OR 2.4, 95% CI 1.1–5.3; *P* = 0.02]. Notably, 37.9% of patients found to have APE were not hypoxic. Patients with positive chest radiographs (CXR) experienced significantly more hypoxia (51.7% (89/172) vs 34.1% (46/135), *P* = 0.002). There was no significant difference between type of PE (saddle, segmental, and subsegmental) and oxygen requirements (*P* = 0.43). There was no significant association between the presence of an infiltrate on CXR and APE (*P* = 0.26).

Median age was observed to be slightly higher in patients with PE (61, IQR 24) compared to 58 (IQR 25), but this difference was not significant (*P* = 0.12). There was no significant difference in body mass index (BMI) between patients who were and were not found to have APE (33, IQR 14 vs 31.5, IQR 11]; *P* = 0.91]. The proportion of patients found to have APE was not significantly different at 0–5, 6–9, and greater than nine days of illness (*P* = 0.83, Table 2). Patients with severe COVID-19 were more likely to have APE (20/145, [13.%] vs 9/263 [3.4%}, OR 4.5, 95% CI 2.0–10.2; *P* < 0.0001). Of the 29 patients with APE, 27 (93.1%) were admitted and followed through their hospital stay.

**Table 2.** Results of computed tomography pulmonary angiogram by day of illness.

Day of illness	Negative APE number (%)	Positive APE number (%)	Total scans
0–5 days	128 (94.1)	8 (5.8)	136
6–9	110 (92.4)	9 (7.6.)	119
>9 days	135 (92.5)	11 (7.5)	146

*APE*, acute pulmonary embolism; *CTPA*, computed tomography pulmonary angiogram.

**Figure 1.**
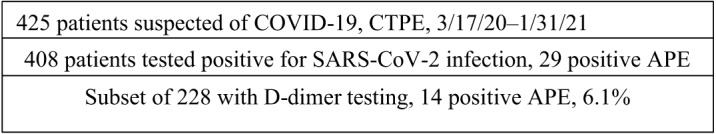
Flow diagram. *COVID-19*, coronavirus disease 2019; *CTPE*, computed tomography pulmonary embolus; *SARS-CoV-2*, severe acute respiratory syndrome coronavirus 2; *APE*, acute pulmonary embolism.

In patients with D-dimer testing, 204/228 (89.4%) were found to have elevated values as defined by local laboratory normal values. In this cohort, detailed in Figure 1, an abnormal D-dimer had a sensitivity of 93% (95% CI 66–100%) and a specificity of 11% (95% CI 7–16%) for the diagnosis of pulmonary embolism. A positive D-dimer was not significantly associated with a diagnosis of APE (*P* = 0.35). Of patients with D-dimer testing and APE, D-dimer values ranged from 134 μg/L to 38,616 μg/L (Table 3). The median D-dimer value was significantly higher in patients who were found to have PE (4,240 μg/L vs 1,030 μg/L; *P* = 0.0048). Patients with a D-dimer of greater than 1,500 μg/L were significantly more likely to have APE (*P* = 0.001).

**Table 3.** Clinical characteristics of patients with D-dimer levels and pulmonary embolism.

D-dimer value (μg/mL)	APE type	COVID-19 severity	Required supplemental O_2_	Oxygen saturation % (triage)	Heart rate (triage)	Day of illness
134	Subsegmental	Severe	No	96	93	7
630	Bisegmental	Less than severe	No	96	94	6
652	Segmental	Less than severe	No	95	102	14
720	Segmental	Severe	Yes	97	99	^**^
1,690	Segmental	Severe	No	99	94	7
3,360	Segmental	Less than severe	Yes	84	117	10
4,200	Subsegmental	Less than severe	Yes	94	97	3
4,280	Segmental	Less than severe	No	95	87	1
4,700	Segmental	Severe	Yes	89	82	11
9,950	Subsegmental	Severe	Yes	95	124	16
11,050	Segmental	Severe	Yes	97	102	12
11,290	Lobar	Severe	Yes	67	111	21
27,412	Segmental	Severe	Yes	93	110	7
38,616	Segmental	Severe	Yes	55	123	5

^**^ Day of illness not documented.

*COVID-19*, coronavirus 2019; *μg/mL*, micrograms per milliliter; *APE*, acute pulmonary embolism.

Using logistic regression analysis in the cohort with D-dimer results, we found that a BMI greater than 32, (OR 4.4, 95% CI 1.0–19.3; *P* = 0.045), a heart rate >90 bpm, (OR 5.0, 95% CI 1.0–24.9; *P* = 0.048), and a D-dimer greater than 1,500 μg/L (OR 5.6, 95% CI 1.6–20.2; *P* = 0.008) were significantly associated with APE. Using a D-dimer cut-off of 1,500 μg/L yielded the best balance of sensitivity and specificity (Table 4) for diagnosis of APE. In patients where D-dimer was obtained, no patients with a D-dimer <1,500 μg/L and a heart rate <90 bpm were found to have APE. Use of these thresholds for CTPA testing would have decreased testing by 27.2%, representing 62 CTPA scans of the 228 patients for whom D-dimers were drawn.

**Table 4.** Test characteristics of different D-dimer cut-points.

D-dimer abnormal cut-point	Chi-square	Sensitivity% (95% CI)	Specificity% (95% CI)
>750 μg/L	0.44	71 (41–92)	39 (32–46)
>1000 μg/L	0.12	71 (41–92)	50 (43–57)
>1500 μg/L	0.001	71 (41–92)	64 (57–71)
>2000 μg/L	0.001	64 (35–87)	77 (71–83)

*μg/L*, micrograms per liter; *CI*, confidence interval.

## DISCUSSION

The primary finding of this multicenter, retrospective cohort analysis showed that the risk factors of BMI greater than 32, a heart rate >90 bpm and D-dimer >1500 μg/L were significantly associated with APE diagnosis in COVID-19 patients. It was interesting that while median BMI did not significantly differ between those with APE and those without APE (33 vs 31.5), a cut-point of >32 was associated with APE. While patients with hypoxia or supplemental oxygen requirement were more likely to have APE, this cohort still showed that a significant portion of APE patients were not hypoxic. The secondary finding shows that D-dimer levels of 1,500 μg/L were significantly associated with APE, while just having a positive D-dimer level was not. The diagnostic yield of APE in our patient population was 7.1%, contradictory to recent literature that showed dramatic increased incidences of APE in COVID-19 patients.^16^ ^,^ ^17^

Striking were the clinical characteristics not statistically significant for association with APE. For example, multiple studies have shown an increased risk of APE in the inpatient setting for patients requiring admission for COVID-19.^18^ This is thought to be related to elevated pro-inflammatory cytokines and abnormalities in coagulation parameters.^19^ We would have expected to find a statistical significance in findings of APE in patients who are later in their illness of COVID-19, after day 6 of illness when symptoms become more pronounced. While we discovered most of our study population (71.4%) was found to have APE after day 5 of COVID-19 illness, this was not statistically significant.

The use of CTPA has associated risks, costs, and staff resources that must be considered when ordering testing. Risks of CTPA include ionizing radiation exposure, contrast nephropathy, and contrast allergies.^20^ ^–^ ^22^ Costs associated with CTPA studies are not just to the patient but to health system capacity, both of which are significant.^23^ In a setting of limited healthcare staffing and bed availability, the increased staff resources required for CTPA studies must be considered.^24^ Thus, reduction in unnecessary CTPA studies would yield multiple benefits.

Our subset of patients for whom D-dimers were obtained (228) offered the best opportunity for reduction in CTPA studies. With almost 90% of our patient population who had D-dimers drawn having an elevated result, using traditional cut-offs were not helpful in evaluation of APE in COVID-19 patients. However, using elevated D-dimer cut-offs in specific patient populations in the evaluation for APE is not a new concept in emergency medicine. The pregnancy-adapted YEARS algorithm and age-adjusted D-dimer cut-off values for diagnosis of suspected APE are two examples of algorithms that use elevated D-dimer value cut-offs.^25^ ^,^ ^26^

We found using a D-dimer cut-off of 1,500 μg/L yielded the best balance of sensitivity and specificity (Table 4) for diagnosis of APE. In patients whose D-dimer was obtained, none of them with a D-dimer <1,500 μg/L and a heart rate <90 bpm were found to have APE. Use of these thresholds for CTPA testing would have decreased CTPA usage by 27.2%, potentially eliminating 62 CTPA studies in the 228 patients for whom D-dimers were drawn.

## LIMITATIONS

Limitations to our study included the inability to quantify clinician gestalt when choosing to order a CTPA study. A second limitation was the inability to use the same D-dimer assay across all hospitals due to different lab equipment. In our study we included two types of D-dimer assays: FEU and DDU. To achieve parity, our methods used standardization and reporting suggested in COVID-19 D-dimer literature review.^15^ While our study covered a regional health system with five EDs, it still lacks generalizability and would require external validation. External validation is particularly important as our study yielded only 14 patients with APE in our D-dimer subset of 228 patients. However, this low yield could potentially be revealing with regard to low overall findings of APE in ED COVID-19 patients.

## CONCLUSION

In patients with acute COVID-19 infections, D-dimer at standard cut-offs was not usable; limiting CTPA using a combination of heart rate >90 bpm or D-dimer >1,500 μg/L would significantly decrease the use of imaging in this population. Future prospective studies are needed to determine whether using this D-dimer threshold and heart rate cut-off in the ED COVID-19 patient population can safely reduce the number of CTPA studies performed.

## Supplementary Information


