# Supplementary material for: Clinical Characteristics of SARS-CoV-2 Acute Pulmonary Embolism and Adjusted D-dimer for Emergency Department Patients
Source: West J Emerg Med. 2023 Oct 25;24(6):1043–8. doi: 10.5811/westjem.58619 (PMC10754201; doi:10.5811/westjem.58619)
Supplement: Supplementary file 1 [file wjem-24-1043-s001.docx]

**Appendix 1:**

COVID related diagnoses:
COVID-19
COVID-19 virus infection
Suspected COVID-19 virus infection
Person under investigation for COVID-19
Lab test positive for detection of COVID-19 virus
Pneumonia due to COVID-19 virus
Educated about COVID-19 virus infection
Advice given about COVID-19 virus infection
COVID-19 virus detected
Acute respiratory disease due to COVID-19 virus
2019 novel coronavirus disease (COVID-19)
Real time reverse transcriptase PCR positive for COVID-19 virus Suspected 2019 novel coronavirus infection
MIS-C associated with COVID-19 (HCC)
COVID toes
Diarrhea due to COVID-19
